# Improved ground-state modulation characteristics in 1.3 μm InAs/GaAs quantum dot lasers by rapid thermal annealing

**DOI:** 10.1186/1556-276X-6-382

**Published:** 2011-05-16

**Authors:** Hanxue Zhao, Soon Fatt Yoon, Chun Yong Ngo, Rui Wang

**Affiliations:** 1 School of Electrical and Electronic Engineering, Nanyang Technological University, Singapore 639798, Singapore; 2 Patterning & Fabrication Capability Group, Institute of Materials Research & Engineering, 3 Research Link, Singapore

## Abstract

We investigated the ground-state (GS) modulation characteristics of 1.3 μm InAs/GaAs quantum dot (QD) lasers that consist of either as-grown or annealed QDs. The choice of annealing conditions was determined from our recently reported results. With reference to the as-grown QD lasers, one obtains approximately 18% improvement in the modulation bandwidth from the annealed QD lasers. In addition, the modulation efficiency of the annealed QD lasers improves by approximately 45% as compared to the as-grown ones. The observed improvements are due to (1) the removal of defects which act as nonradiative recombination centers in the QD structure and (2) the reduction in the Auger-related recombination processes upon annealing.

## Introduction

Quantum dots (QDs) are promising for realizing fast and stable laser sources in fiber optic applications, due to their superior characteristics over conventional quantum well (QW) lasers, such as low threshold current, high internal efficiency, and infinite characteristic temperature [1,2]. However, the high-speed performance of QDs is generally poorer than that of QWs due to several factors: slow inter-level relaxation of the carriers [3], finite density of state (DOS), and closely spaced hole energy levels [4]. Slow carrier relaxation rate, in combination with the limited DOS, will lead to early switching from ground-state (GS) lasing to excited-state (ES) lasing at high temperature or at high drive current. This is undesired since ES lasing reduces GS lasing efficiency due to gain saturation of the GS transition. This, due to state filling effect in discrete quantum levels, degrades the high-speed characteristics [2,5] of the QD lasers. Furthermore, for high-speed modulation, shorter cavity length is favored. Unfortunately, transition from GS to ES lasing occurs earlier in short cavity lasers (≤1 mm) due to the increased cavity loss as compared to long cavity lasers [3]. Thus, to improve the high-speed performance of QD lasers, it is important to delay the onset of ES lasing. It is well-documented that *p*-doping of the QDs can reduce the effect of gain saturation and thus maintain GS lasing up to higher operating temperatures [1], and potentially faster relaxation time [6]. On the other hand, rapid thermal annealing (RTA) might result in intermixing of the QDs with the surrounding matrix and tunable inter-level energy. This may lead to faster inter-level relaxation of the carriers [7], and consequently, suppression of ES lasing and improved high-speed modulation efficiency. However, there exists only a handful of works on the effects of RTA on GS modulation of the annealed *p*-doped QD lasers [8-10].

In this work, we have investigated the effect of RTA on the GS modulation characteristics of 1.3 μm *p*-doped InAs/GaAs QD lasers. Both the modulation bandwidth and efficiency were found to increase significantly upon annealing.

## Experimental details

The ten-layer self-assembled *p*-doped InAs/GaAs QD laser structure used in this experiment was grown on GaAs (100) substrate by molecular beam epitaxy (MBE). The structure consists of QD active region sandwiched between two 1.5 μm C- and Si-doped Al_0.35_Ga_0.65_As cladding layers. The active layer comprises 2.3 monolayer (ML) of InAs QDs capped by a 5-nm In_0.15_Ga_0.85_As layer. A 33-nm GaAs layer is used to separate the two QD layers. P-doping modulation (~16 acceptors per QD) was incorporated into the 10 nm GaAs layer in the middle of each 33 nm-thick spacer between the QD rows [11]. The indium-containing layers were grown at approximately 485°C, while the (Al)GaAs layers were grown at approximately 580°C. The QD samples were capped with 200 nm of SiO_2 _deposited by plasma-enhanced chemical vapor deposition (PECVD) before annealing. The annealing process was then performed in N_2 _ambient at 600°C for 15 s using a rapid thermal processor. The choice of annealing conditions was determined from our recently reported results [9]. Subsequently, the as-grown and annealed samples were processed into 4-μm-wide narrow ridge waveguide lasers [12]. The high-speed modulation of the as-cleaved QD lasers was performed under continuous-wave (CW) biasing condition using a vector network analyzer (VNA) and a high-speed photoreceiver [13]. The spontaneous emission intensity of the device under different bias currents was obtained with an integrating sphere. A thermoelectric temperature controller controls the device temperature during measurements.

## Results and discussion

**Figure 1:**
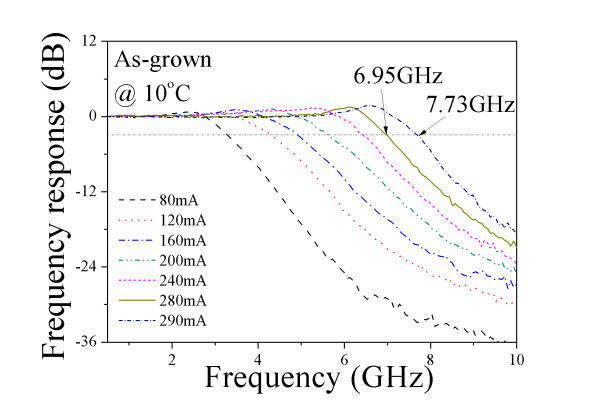
**The frequency response of a 1-mm long as-grown QD laser measured at 10°C**.

**Figure 2:**
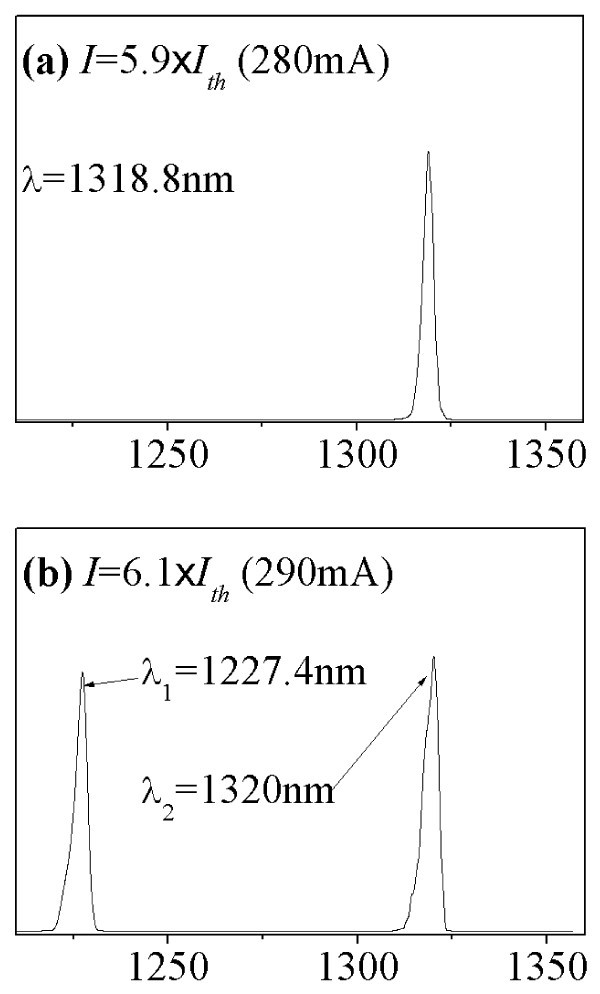
**The lasing spectra of the same as-grown QD laser at 10°C measured at (a) 280 mA, and (b) 290 mA**.

Figure 1 shows the frequency response of the as-grown device with cavity length of 1 mm, measured at 10°C. The threshold current (*I*_th_) of the as-grown device is approximately 51.6 mA. The bandwidth increases gradually with bias currents. The maximum bandwidth obtained is 7.73 GHz. Figure 2 shows the lasing spectra of the same as-grown QD laser at 10°C measured at (a) 280 mA, and (b) 290 mA.. Note that the ES lasing threshold (*I*_th,ES_) of the as-grown device is 284 mA. At the bias current of 5.9 × *I*_th _(280 mA), the laser emission wavelength is approximately 1319 nm and corresponds to the GS lasing (refer to Figure 2a). When increasing the bias current to 6.1 × *I*_th _(290 mA), emission wavelengths of approximately 1227 and 1320 nm exist simultaneously (refer to Figure 2b). The energy separation between the two states is approximately 71 meV. Thus, in the as-grown laser, GS lasing dominates at low bias currents, while ES lasing dominates at higher bias currents. The bandwidth of 7.73 GHz for the as-grown device measured at 290 mA includes the contribution from ES lasing. The bandwidth obtained from purely GS lasing is 6.95 GHz at 280 mA. Slight increase of the bias current by 10 mA causes a significant increase of bandwidth by 0.78 GHz (from 6.95 to 7.73 GHz), which is likely due to the additional photons emitted by the ES lasing. However, the bandwidth contributed by ES lasing is undesired since the wavelength of interest for telecommunication is 1.3 μm.

**Figure 3:**
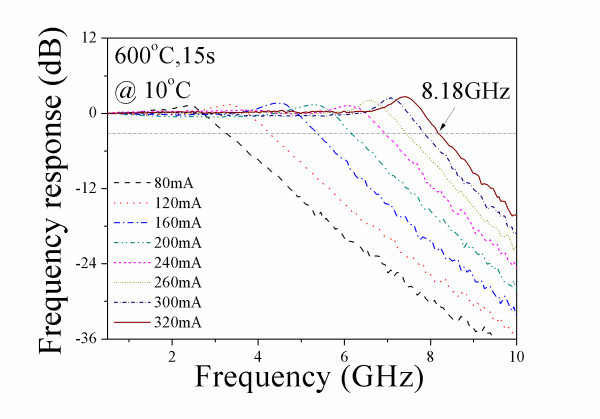
**The frequency response of a 1-mm long 600°C annealed QD laser measured at 10°C**.

**Figure 4:**
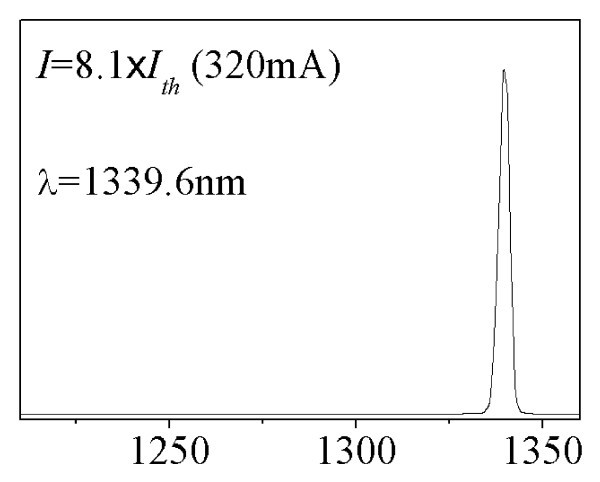
**The lasing spectrum of the same annealed QD laser measured at 320 mA**.

**Figure 5:**
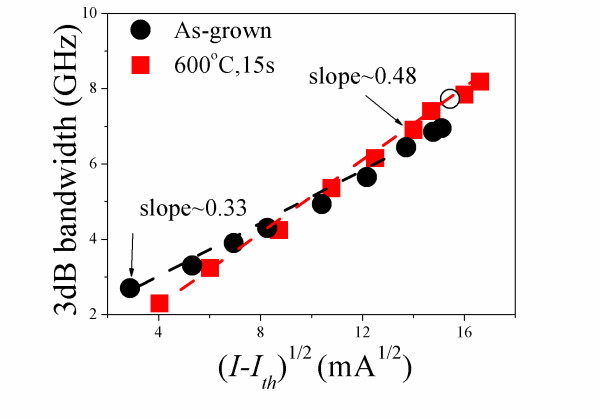
**The measured bandwidth as function of normalized bias current (*I-I***_**th**_**)**^**1/2 **^**measured at 10°C for the: as-grown QD laser GS emission indicated with solid circles, annealed QD laser GS emission indicated with solid squares, and as-grown QD laser ES emission indicated with hollow circles**.

Figure 3 shows the frequency response for the 600°C annealed device with cavity length of 1 mm, at 10°C. The *I*_th _of the 600°C annealed device is approximately 42.6 mA. The lasing spectrum of the 600°C annealed device at 8.1 × *I*_th _(320 mA) is shown in Figure 4. Since the bandwidth saturates at current greater than 320 mA, the maximum bandwidth obtained is 8.18 GHz. After annealing, 18% improvement in modulation bandwidth has been achieved. It is worth mentioning that no ES lasing has been observed in the annealed laser for all bias current levels. Therefore, the maximum bandwidth of 8.18 GHz for the 600°C annealed device is purely from GS lasing. Comparing Figure 2a and Figure 4, the difference in the GS wavelength might be due to the carrier induced bandgap shrinkage [14] and the thermal induced emission wavelength shift [15-17]. At a fixed current injection above threshold, a higher *I*/*I*_th _ratio is resultant in the annealed lasers (8.1 × *I*_th_) than in the as-grown lasers (5.9 × *I*_th_) due to the reduction of threshold current after annealing [13]. The thermal effects may become more dominant in affecting the wavelength due to annealing. The reasons of this wavelength shift are currently under investigation. Figure 5 shows the measured bandwidth as function of normalized bias current (*I-I*_th_)^1/2 ^measured at 10°C for the: as-grown QD laser GS emission indicated with solid circles, annealed QD laser GS emission indicated with solid squares, and as-grown QD laser ES emission indicated with hollow circles. The slope deduced from the bandwidth vs. (*I-I*_th_)^1/2 ^(also known as modulation efficiency, *η*_MOD_) is approximately 0.33 GHz/mA^1/2 ^for the as-grown device at low bias. Note that there is a sudden increase of the *η*_MOD _when ES lasing starts to dominate. GS lasing dominates in the 600°C annealed device throughout the measured bias current range, while ES lasing has been completely suppressed, even at higher bias currents. The *η*_MOD _for the 600°C annealed device is approximately 0.48 GHz/mA^1/2^. Note that the *η*_MOD _has been increased by approximately 45% after the 600°C RTA process. Shi et al. [3] have shown that fast inter-level relaxation is favorable for the GS lasing, and leads to higher QD GS capture efficiency. When the inter-level relaxation becomes slower, carriers captured into the ES cannot relax to the GS immediately and continues to occupy the ES. The ES lasing will occur when the population inversion for the transition between the ES electrons and the GS holes is satisfied. Higher *η*_MOD _in the 600°C annealed laser indicates lower wetting layer carrier occupation probability and higher GS capture efficiency, which may be due to faster inter-level relaxation induced by RTA [9].

**Figure 6:**
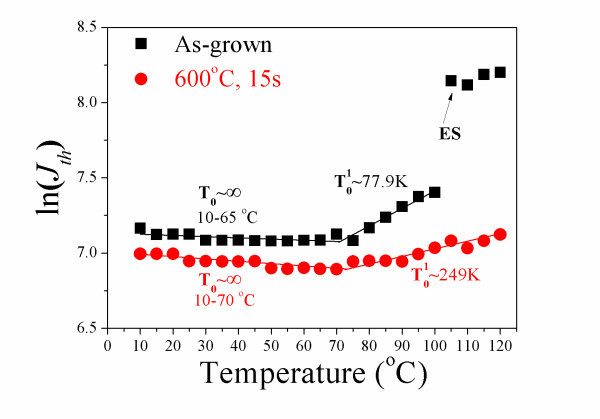
**Plot of ln(*J***_**th**_**) vs. temperature of the as-grown (square) and 600°C annealed (circle) QD lasers**.

Figure 6 shows the logarithmic values of threshold current densities (ln(*J*_th_)) of as-grown and 600°C annealed devices measured as function of temperature. The as-grown and 600°C annealed lasers exhibit infinite *T*_0 _at temperature range of 10-65 and 10-70°C, respectively. The infinite *T*_0 _could be explained by the increase in Auger recombination and improved carrier thermalization with increasing temperature. The former will increase the threshold current, while the latter decreases the threshold current by increasing the efficiency of the radiative recombination due to thermal escape of carriers from the dots and transfer into deeper levels (larger dots) [1]. Due to *p*-type modulation doping in the QDs, the electrostatic attraction of the excess holes increases the effective barrier for electron escape, thus limits the electron thermalization till higher temperature as compared to the intrinsic device and results in a larger *T*_0 _[18]. The as-grown laser exhibits *T*_0_^1 ^~ 77.9 K from 70 to 100°C. ES-dominated lasing starts at 105°C in the as-grown laser. However, in the annealed laser, GS lasing dominates throughout the measured temperature range, up to 120°C. The annealed laser exhibits *T*_0_^1 ^~ 249 K from 75 to 120°C. As shown in Figure 6, as compared to the as-grown lasers, the threshold current has been reduced and *T*_0_^1 ^has been improved for the annealed QDs up to 120°C. These are possibly due to the reduction in Auger recombination in the annealed QDs since Auger recombination increases the threshold current. In agreement with the work of Shchekin et al. [19], the modulation response of QD laser is closely related to the threshold characteristics. Although QDs have been predicted to be modulated at very high frequencies theoretically, it has been shown by Deppe et al. [4] that the current densities required to reach these values should be considered. The low threshold current density of the annealed QD laser suggests that this device can be driven far above threshold and reaches higher speed. It has been pointed out by Marko et al. that Auger recombination is an important loss process in 1.3 μm QD lasers around room temperature [18]. *p*-Doping of the QDs enhances the gain, but it also increases the threshold current, possibly due to increased Auger recombination [20]. The improved *T*_0_, together with the reduced threshold current density in the annealed lasers, implies that the severe nonradiative Auger recombination is possibly reduced as a consequence of thermal annealing by reducing the defect densities. And this potential results in improved carrier relaxation, thus improved high-speed modulation characteristics.

**Figure 7:**
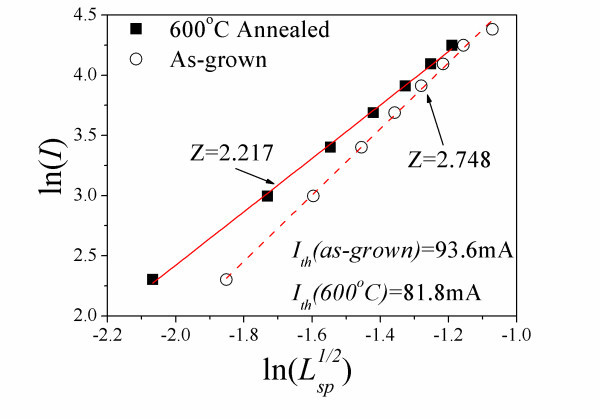
**Plot of ln(*I*) vs. ln(*L***_**sp**_^**1/2**^**) for the as-grown and 600°C annealed QD devices with cavity length of 2 mm at room temperature**. The *I*_th,GS _of the as-grown and 600°C annealed QD device is 93.6 and 81.8 mA, respectively.

Figure 7 shows the plot of ln(*I*) vs. ln(*L*_sp_^1/2^), where *I *is the injection current and *L*_sp _is the total spontaneous emission intensity. The gradient obtained is known as the *Z *value. The total spontaneous emission intensity is measured by modulating the device in the unamplified region, where *I < I*_th_. Over this limited range of *I*, we employed a general relationship between *I *and *L*_sp _[21]: ln(*I*) ∝ *Z *ln(*L*_sp_^1/2^), where 1 ≤ *Z *≤ 3, depending on the relative importance of nonradiative monomolecular recombination process due to defects or impurities (*Z *= 1), bimolecular radiative recombination process (*Z *= 2) or nonradiative Auger recombination process (*Z *= 3). Generally, 2 ≤ *Z *≤ 3 applies in InAs/GaAs QDs, especially in *p*-doped QDs, due to the enhanced nonradiative Auger recombination [22]. As shown in Figure 7, *Z *reduces from 2.748 for the as-grown device to 2.217 for the annealed device. This suggests that the radiative recombination becomes more dominant than the Auger-related recombination processes resulting from annealing [21]. It is thus reasonable to say that the radiative recombination has been enhanced after 600°C RTA. The suppression of nonradiative Auger recombination may also result in smaller carrier lifetime, thus improved high-speed modulation performances, which agrees well with our experimental results on the improved small signal modulation performances in annealed lasers (as shown in Figures 1, 3, 5). Furthermore, our experimental results also demonstrate improved quantum efficiency and reduced optical loss in the 600°C annealed lasers, which have been submitted elsewhere [9].

## Conclusions

In summary, we have investigated the GS modulation characteristics of 1.3 μm InAs/GaAs QD lasers that consist of either as-grown or annealed QDs. Compared to the as-grown QD lasers, the annealed ones exhibit approximately 18% improvement in the modulation bandwidth and approximately 45% in the modulation efficiency. The observed improvements are due to the reduction in the Auger-related recombination processes and the removal of nonradiative recombination centers in the QDs upon annealing.

## Abbreviations

CW: continuous-wave; DOS: density of state; ES: excited-state; GS: ground-state; MBE: molecular beam epitaxy; ML: monolayer; *PEVCD*: plasma-enhanced chemical vapor deposition; *QD*: quantum dot; *QW*: quantum well; *RTA*: rapid thermal annealing; *VNA*: vector network analyzer.

## Competing interests

The authors declare that they have no competing interests.

## Authors' contributions

ZHX: conceived of the study, participated the device fabrications, carried out the static and small signal characterizations, performed the experimental analyses, and drafted the manuscript, YSF: conceived of the study, and participated in its design and coordination, CYN: performed the Rapid Thermal Annealing, and participated in the design and coordination of the study, WR: conceived of the study, participated in its design and coordination, and participated the device fabrications. All authors read and approved the final manuscript.
